# Treatment of benign bone lesions with an injectable biphasic bone substitute

**DOI:** 10.1186/s12891-022-05843-3

**Published:** 2022-10-08

**Authors:** Kevin Döring, Colleen Rentenberger, Alexander Kolb, Janina Patsch, Stephan Puchner, Reinhard Windhager, Catharina Chiari

**Affiliations:** 1 Department of Orthopedics and Trauma Surgery, Medical University of Vienna, Vienna General Hospital, Währinger Gürtel 18-20, A-1090 Wien, Österreich; 2 Division of General and Pediatric Radiology, Department of Biomedical Imaging and Image-guided Therapy, Medical University of Vienna, Wien, Austria

## Abstract

**Background:**

Injectable biphasic ceramic bone substitutes (BCBSs) represent a modern alternative to conventional options for bone defect filling, as they further open the possibilities for percutaneous cavity reconstruction. Although recent studies have shown good surgical outcomes after treatment with BCBSs, mid-term follow-up data are still missing.

**Patients and methods:**

Between 2013 and 2017, 18 patients were [1] treated with BCBS [2] for benign bone lesions and [3] had a complete set of retrospective information, including surgical protocols, imaging, patient dismission letters and outpatient clinic protocols, [4] with a minimum follow-up time of one year. Eleven patients received percutaneous surgery, while 7 patients had open curettage and BCBS filling. The median follow-up time was 36.5 (range 12–80) months.

**Results:**

Local recurrence was reported in four patients. A distinctive bone remodelling pattern was noted on follow-up X-ray and magnetic resonance imaging showing a double-line phenomenon and continuously increasing cortical thickness one year after treatment in nine of thirteen patients. Regarding surgical complications, one patient suffered from a septic complication that required BCBS removal and lavage. One patient experienced superficial surgical site inflammation with redness and swelling, while two other patients had prolonged wound secretion.

**Conclusion:**

In a limited case series, the studied BCBS demonstrated acceptable surgical outcomes. Initial wound leakage and recurrence seemed to be associated with percutaneous injection. Further studies are needed to compare recurrence and bone graft resorption after open and percutaneous bone cyst surgeries and to further evaluate postoperative surgical site inflammation, which appears self-limiting in most cases.

## Introduction

In many institutions, the standard therapeutic approach for the operative treatment of benign bone tumours and tumour-like lesions consists of open surgical intralesional curettage with or without the application of adjuvant treatment methods, such as cavity burring with a mechanical burr, phenolization, sclerotherapy or cryotherapy, to further reduce potentially remaining tumour cells. [1–4] The remaining bone cavity is usually filled and impacted with either autograft bone, with iliac crest autograft as a widely accepted gold standard, or an allograft, such as bone chips or bone substitute materials, to circumvent the possible donor site morbidities and quantity limitations associated with autograft bone retrieval. Calcium phosphates and calcium sulfates are widespread bone substitute materials for defect reconstruction. [5–8] Calcium phosphates, such as hydroxyapatite (HA), serve as osteoconductive porous scaffolds to enhance cell migration and bone formation while providing structural support. HA can be optionally ground to powders and combined with other bioactive materials to further improve bone mineralization. [8] In this context, faster dissolving calcium sulfates are known for their osteoinductive behaviour and can serve as resorbable carriers for calcium phosphates. [9, 10] A general trend towards minimally invasive procedures can be observed in all surgical disciplines to diminish the risks of open surgical treatment, such as surgical site infections, pain and functional impairment. In some benign bone tumours, this trend has resulted in the implementation of biopsies with an intention to cure (“curopsy”), percutaneous agent instillations, or even noninvasive treatments, such as medication with bisphosphonates or denosumab. [11–18] Although these minimally invasive techniques have shown acceptable results regarding recurrence in recent literature, there remains a postoperative bone cavity of variable dimensions.

Novel galenic formulations have been introduced to accommodate the surgical needs of defect reconstruction. In this context, biphasic ceramic bone substitute (BCBS) powders consisting of HA and calcium sulfate have a potential application in the minimally invasive treatment of benign and borderline bone tumours, as BCBSs can be moulded and injected into tumour cavities after the treatment of cystic lesions. BCBSs have been successfully used in the reconstruction of bone defects after the surgical treatment of benign bone tumours or to fill the defects of depressed metaphyseal or comminuted fractures. [19–23]

Although earlier studies showed that BCBSs had a good remodelling effect, follow-up data regarding bone graft resorption and consolidation after BCBS treatment are sparse. Additionally, there are no magnetic resonance imaging (MRI) follow-up data describing BCBS ingrowth aside from a single case report. [24]

We therefore asked:

1) What is the treatment failure rate after surgical treatment with a BCBS?

2) How does the cavity morphology change in the postoperative course?

3) What are the treatment-specific complications?

## Patients and methods

After receiving approval from the local ethics committee, a retrospective analysis of the data in the in-house software system (“AKIM”, Siemens) identified 21 patients. After application of the inclusion criteria, the included patients [1] received treatment with a BCBS [2] for benign bone tumours or tumour-like lesions 3) and had a complete set of retrospective information, including surgical protocols, X-rays, patient dismission letters and outpatient clinic protocols, [4] with a minimum follow-up of one year; two patients were excluded because they were followed up for less than one year, and one patient was excluded because of an inconclusive histological report. Thus, the analysed population consisted of 18 patients. Thirteen patients were male, and 5 patients were female. The median patient age was 14 (range 6–25) years. Histologically, the tumour entities were unicameral bone cysts (UBCs, n = 13), aneurysmal bone cysts (ABCs, n = 2), secondary ABC (n = 1), and nonossifying fibroma (NOF, n = 2). The bone tumours were primarily treated with a BCBS in 7 cases, and in 11 cases, a BCBS was used to treat recurrent bone tumours. The median follow-up time was 36.5 (range 12–80) months. Further basic demographic information is depicted in Table 1.

**Table 1:** Demographic parameters

Parameter	Patients (n = 18)	
Median age at index surgery	14 years (range 6–25)	
Median follow up time	36.5 months (range 12–80)	
Median time to recurrence after index surgery	9.5 months (range 4–51)	
**Sex**		
Male/Female	13/5	
**Localization**		
Humerus	13	
Femur	4	
Tibia	1	
**Imaging**		
Median cyst volume	27ml (range 10–63)	
Soft tissue leakage	6	
**Tumor entities**		
Unicameral bone cyst	13	
Aneurysmal bone cyst	2	
Non-ossifying fibroma	2	
Secondary aneurysmal bone cyst	1	
Recurrent lesion	11	
**Surgical procedure**		
Percutaneous treatment	11	
Curettage	7	
Median amount BCBS delivered	18ml (range 10–38)	

**Fig. 1:**
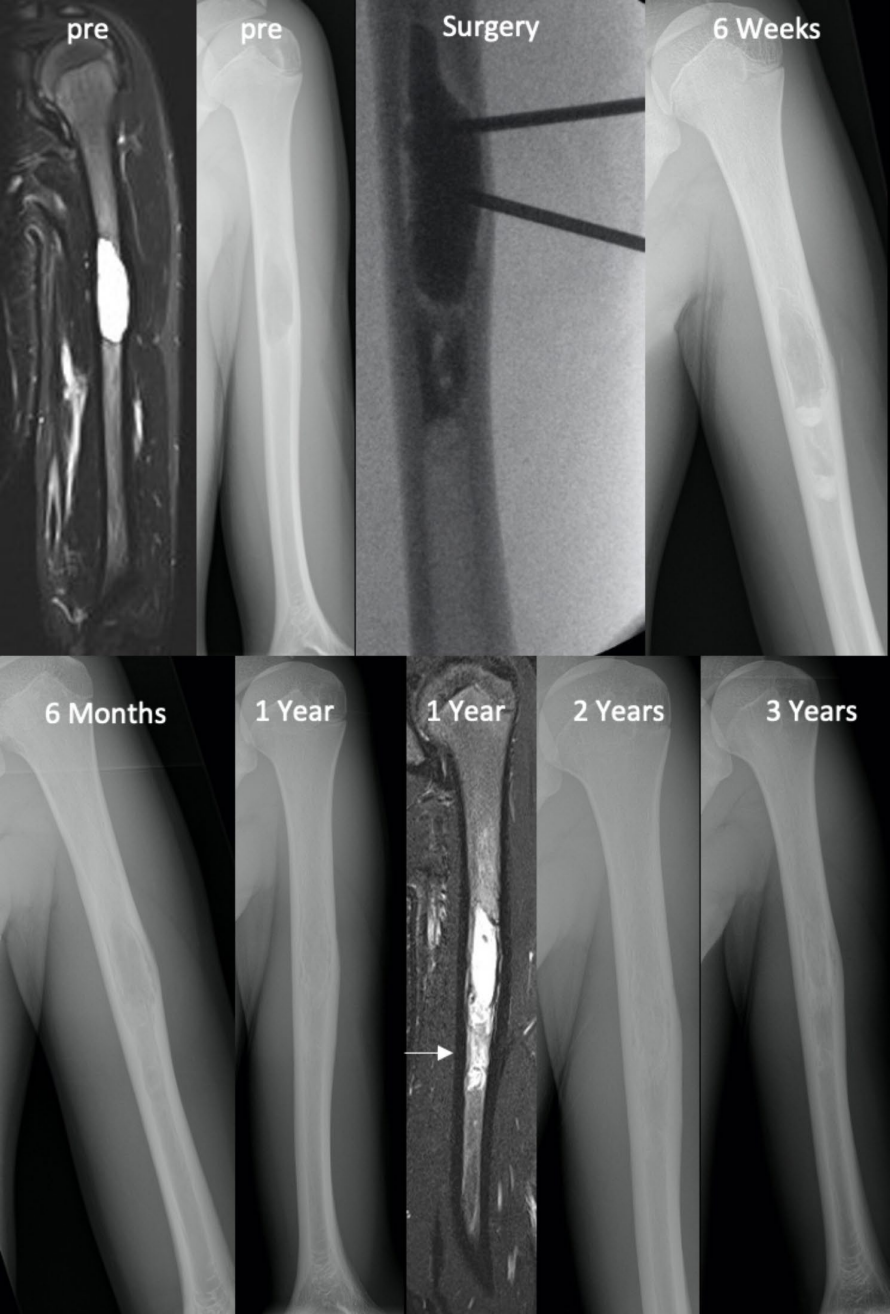
A 15-year-old male patient with a recurring unicameral bone cyst after two open surgeries with curettage and filling with allograft bone chips six years and two years before index surgery. During index surgery, the cyst was percutaneously filled using a two-needle technique. On postoperative X-rays, contrast levels in the distal regions of the cavity could be observed six weeks after surgery, and a double-line phenomenon was visible in the rim area. On MRI one year after surgery, a T2 hyperintense signal with a cylindric filling core could be observed (white arrow). Gradual bony consolidation was found on follow-up X-rays, with full consolidation two years after index surgery

In the first postoperative year, clinical check-up visits with X-ray imaging were performed after 6 weeks, 3 months, 6 months and 12 months and every 6 months thereafter. The X-ray images were screened for contrast levels, BCBS residua in the soft tissue, recurrent bone tumours and the “double line” phenomenon in cortical bone. This double line phenomenon consisted of a duplication of the cavities’ border shadow and was typically observed on postoperative X-ray images. (Fig. 1) Morphologic cavity changes were graded according to the modified Neer classification. [25] MRI was primarily used for patients with suspected recurrence, incomplete cavity consolidation or BCBS resorption. Twelve patients received a first MRI with a median of 12.5 (range 6–45) months after surgery, while four of these patients received a follow-up MRI 29.5 (range 13–44) months after surgery.

## Surgical procedure

Either percutaneous or open surgery was performed before filling with a CERAMENT Bone Void Filler (BONESUPPORT, Lund, Sweden); these procedures were conducted by seven orthopaedic surgeons in this study. In percutaneous treatment, cyst localization was confirmed with mobile X-ray image intensifier before stab incision and preparation to the bone. Cystic lesions were opened with two Jamshidi needles. The first Jamshidi needle was placed in the distal portion of the tumour, while the second needle, which served as a “ventilation needle”, was placed in a more proximal cyst localization and at a different angle. The cysts were then washed with saline before the cyst walls were scraped with K-wires, which were inserted over the Jamshidi needles. This technique was further promoted by Kaczmarczyk et al., who showed that cyst wall scraping was sufficient to allow for bony ingrowth in the allograft composite. [23] An adequate amount of BCBS was prepared and applied over the distal needle under an X-ray image intensifier. The proximally placed needle was closed when the BCBS drained over the needle opening. After X-ray confirmation of thorough lesion filling, a ten-minute waiting interval was observed to ensure BCBS hardening.

In open surgery, after approaching the bone, either an adequately sized bone flap was lifted off the cyst wall or, when osteosynthesis removal was indicated, the recurrent cyst was approached over the remaining screw tunnel. Afterwards, the cavity was cleared of tumour masses and septa via curettage. As adjuvant treatments to open surgery, one patient received cavity burring with a mechanical burr, while another patient received additional phenolization using phenol-soaked swabs after curettage. Thereafter, the inside of the cavity was filled with BCBS. After a standard ten-minute interval to await BCBS hardening, the wound was closed.

### Statistical analysis

Statistical analyses focused on treatment outcomes after BCBS insertion. Descriptive statistics were applied to depict frequencies, means and ranges of relevant parameters. Revision surgeries included those for recurrences, defined as progressive lesions in a previously obliterated area as described by the modified Neer classification system, and surgical site complications. [25] The follow-up duration was calculated from the date of surgery with BCBS filling to the date of the last follow-up visit. Revision intervals were calculated from the date of surgery with BCBS filling to the date of revision surgery. The median preoperative cavity volume was calculated using preoperative MRI. Statistical calculations were made using SPSS version 26 (IBM).

## Results

Recurrent bone tumours were observed in 4 patients who underwent percutaneous treatment. The recurrent lesions were one ABC, one secondary ABC and two UBCs. Revision surgeries due to recurrent bone tumours were performed after a median time of 9 months (range 4–51) after initial BCBS treatment. Three of these four patients were treated with open curettage and filling with allograft bone chips, while one patient received percutaneous sclerotherapy. (Table 2) Percutaneous surgeries were performed in 11 patients, while open curettage and filling were performed in 7 patients. The median BCBS filling volume was 18 (range 10–38) ml. No pathologic fractures occurred after BCBS treatment.

**Table 2:** Overview of reoperations

Patient	Localisation	Histology	Percutaneous	Indication	Treatment	Time
1	Humerus	ABC	1	Recurrence	Curettage, Phenolization, Allograft	4 months
2	Humerus	UBC	0	Septic revision	Filling material extraction	2 weeks
3	Humerus	sABC	1	Recurrence	Curettage, filling with allograft	9 months
4	Femur	UBC	1	Recurrence	Curettage, filling with allograft, osteosynthesis	51 months
5	Humerus	UBC	1	Pain	Material removal (Titanium elastic nail)	21 months
6	Humerus	UBC	1	Recurrence	Percutaneous sclerotherapy	10 months

ABC = aneurysmal bone cyst, UBC = unicameral bone cyst, FD = fibrous dysplasia, sABC = secondary aneurysmal bone cyst

**Fig. 2:**
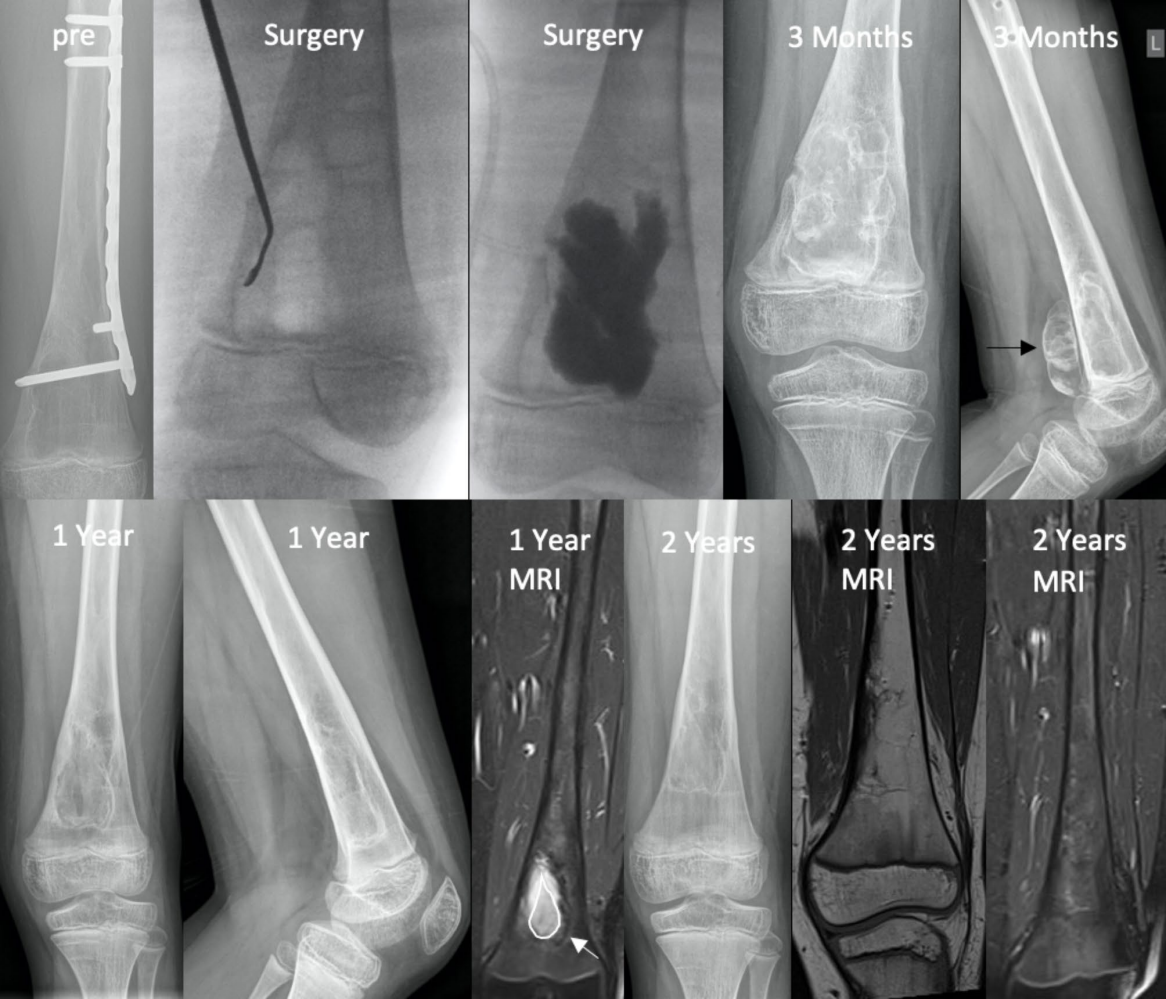
A 10-year-old male patient who suffered from a pathologic fracture due to a unicameral bone cyst and consequently received open curettage, filling with allograft bone chips and osteosynthesis. (Pre) Recurrence was visible two years after surgery, which led to osteosynthesis removal, open curettage and filling with a BCBS. (Surgery) On early postoperative X-rays, residual BCBS in the popliteal soft tissue (black arrow) could be observed, which gradually vanished by one year after surgery. On MRI one year after surgery, a typical inhomogeneous T2 hyperintense transformation zone (white arrow) and a teardrop-shaped filling core (white circle) could be seen, which faded with bony integration on follow-up MRI two years after the index procedure

**Fig. 3:**
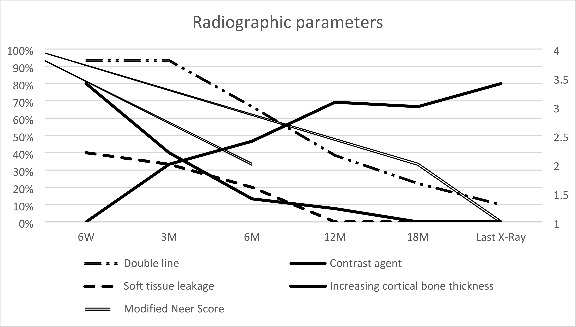
Radiographic parameters at postoperative check-up visits Left: Percentage of patients with different radiographic findings Right: Median modified Neer scores

Twelve months after treatment, 2 out of 17 cavities showed full resolution (Neer I), and 7 cavities showed partial resolution (Neer II). Persisting lesions (Neer III) were observed in three patients, while three other patients suffered from recurrences (Neer IV) in the first postoperative year. One patient underwent BCBS extraction prior to completing a twelve-month follow-up period. X-ray examinations revealed BCBS leakage in 6 patients, with the BCBS residua in the soft tissue completely vanishing in the first postoperative year in all cases. (Fig. 2) Nine out of thirteen patients showed an increased cortical bone thickness on imaging one year after treatment. The modified Neer score decreased accordingly, with nine out of thirteen lesions classified as “healed” or “healing with defect” and a median Neer score of 2 (range 1–4) one year after surgery. BCBS insertion resulted in a characteristic radio-opaque “double line” around the border of the bone cavity on early X-rays. The double-line phenomenon was observed in fourteen of fifteen patients three months after surgery and vanished in all but two patients by 18 months after surgery. A radiopaque fluid in the distal part of the cavity, corresponding to the mixture of the iohexol contrast agent with the BCBS, was visible in twelve of fifteen patients six weeks after surgery and quickly vanished thereafter. (Fig. 3)

On initial MRIs acquired 12.5 (range 6–45) months after surgery, a T2 hyperintense and T1 hypointense cavity signal with sclerotic cavity borders and a central filling material core were typically seen. This observation corresponded to the status of the bone substitute before full remodelling. Progressive bony consolidation was seen in three of four follow-up MRIs, at a median of 29.5 (range 13–44) months after surgery. In all these cases, no complete consolidation was observed.

Regarding revisions and major surgical complications, the BCBS was removed during revision surgery in one patient. The patient suffered from a septic complication and received BCBS extraction and lavage. (Table 2)

Regarding minor surgical complications, one patient experienced superficial postoperative surgical site inflammation with redness and swelling, while two other patients suffered from wound secretion after percutaneous BCBS insertion. Only one patient suffering from wound healing complications had BCBS residua in the soft tissue on X-ray examinations.

## Discussion

Percutaneous treatment of benign bone neoplasms is a promising alternative to standard open curettage and filling. In this context, only injectable filling materials can be used to access bone cavities after percutaneous surgery. CERAMENT Bone Void Filler, as a mouldable and injectable calcium sulfate and HA bone substitute combination, showed good outcomes in terms of defect filling and recurrence rates in previous studies. [10, 22] As previous literature only showed short-term follow-up outcomes with mean periods ranging from 12 to 22 months [10, 22, 23], little is known about the middle- to long-term follow-up outcomes. We found that the BCBS showed acceptable recurrence rates in benign bone lesions at a median follow-up of 3 years. We further observed distinctive degradation and remodelling behaviour of this material, with progressive defect healing and increasing cortical bone thickness one year after surgery in most cases. This information should promote the use of percutaneous surgery and filling with injectable bone substitutes, especially in recurrent benign lytic lesions with confirmed tumour histology.

## Revisions after surgical treatment

The treatment failure rate was acceptable, with 5 out of 18 patients experiencing surgical revision due to treatment failure and four of these patients suffering from recurrent lesions. We found that all patients suffering from local recurrences underwent percutaneous surgery. Percutaneous and open surgery are established surgical strategies for the treatment of benign lytic bone lesions. Open curettage can lead to acceptable recurrence rates through manual tumour cell removal and cyst lining disruption, as well as potential access to growth factors and stem cells by entry into the medullar cavity. [26, 27] However, because of the invasiveness and associated complications of open procedures, some authors do not recommend initial open surgery for some benign bone lesions. [28] In percutaneous treatment approaches, various sclerosants, such as polidocanol, ethanol or doxycycline, may be used in addition to mechanical cyst membrane disruption. [23, 29, 30] In a larger case series, Rastogi et al. observed a lesion size reduction of 76.6% after a mean of 3 injections in 72 patients with ABCs treated with polidocanol [31], while Marie-Hardy et al. reported a lesion size reduction in 85% in 55 ABCs treated with 96% ethanol in a mean of 1.7 injections. [32] We think that additional cavity reconstruction using injectable bone void fillers may be of particular benefit for minimally invasive treatments of recurrent benign bone lesions to further promote bone healing.

To put our findings into context with studies using the same BCBS, Kotrych et al. showed a very low recurrence risk with no reported recurrence in 33 patients after open curettage and filling of benign bone tumours, although the short median follow-up of 10 months needs to be mentioned. [10] In a prospective case series of 14 patients with solid and cystic benign bone tumours, Kaczmarczyk et al. reported no recurrence at the 12-month follow-up after treatment. [23] To date, studies analysing bone remodelling and recurrence after the treatment of benign bone lesions with BCBS suffer from high heterogeneity in surgical approaches and tumour entities. Larger and more homogenous case series are needed to evaluate the clinical value of these novel filling materials.

## Bone remodelling behaviour

On postoperative X-ray and MRI, the BCBS showed a characteristic remodelling behaviour, with a distinctive double-line phenomenon at the cavity borders and progressively increasing cortical bone thickness in the months after treatment. (Fig. 1) In this context, the inherent microporosity of the material allows for direct penetration of tissue fluids and thus cells and growth factors after implantation, which promotes the natural bone healing process. [23] However, the material properties also allow for postoperative soft tissue leakage, which was observed in six patients in this case series. Although the shrinking of the residual BCBS in the soft tissue should be monitored by X-ray, there is no evidence of a higher chance of postoperative soft tissue inflammation in these patients. [22]

MRI was used in cases of suspected recurrence or incomplete lesion consolidation in this study. In these cases, we did not find full bony consolidation but instead a central bone substitute core with an inhomogeneous T2 hyperintense transformation zone at the cavity borders. Further X-ray examinations at regular follow-up intervals showed complete cavity remodelling 9–18 months after surgery. This gradual degradation is a known feature of different multiphasic injectable bone substitutes, which may show barrier-like structures at the surface that control connective tissue ingrowth into the central regions of the implantation bed. [33, 34] This characteristic may allow for guided bone regeneration with bone formation directed from the defect barriers into the central defect regions. [33]

A previous case series on BCBS demonstrated the healing process on follow-up X-ray and computed tomography images, showing peripheral reactive zones, trabecular bone bridging and improved cortical bone thickness one year after surgery. [22, 23] We think that both MRI and computed tomography have different advantages in evaluating bone healing and suspected recurrences, but MRI avoids radiation exposure and thus should be recommended, especially in a presumably young patient population.

## Surgical complications

Complications after BCBS application mainly consisted of superficial inflammation and secretion. One patient needed revision surgery due to wound breakdown and secretion. A primarily sterile inflammatory response is a known complication of calcium sulfate grafting. [35–37] Although it may be assumed that the inflammatory processes are caused by rapid graft resorption and thus calcium-rich fluids in surrounding soft tissue, which lead to a decrease in the local pH value and an invasion of inflammatory cells [38, 39], Nilsson et al. showed that carbonated HA precipitates on the implant surface, which may allow for a more gradual dispersion and resorption of calcium sulfate in a calcium sulfate and HA biocomposite. [40] This gradual resorption is a desirable trait for bony ingrowth, as residual HA provides stability to the newly mineralizing tissues. [41]. The presence of a local inflammatory response and secretion after BCBS insertion is inconsistently described in the literature. McNally et al. observed fewer prolonged wound leakages and lower recurrence rates with a ceramic biphasic bone substitute with gentamicin than with a collagen fleece with gentamicin or calcium sulfate with tobramycin pellets for dead space filling after resection of chronic osteomyelitis. They attributed their results to higher antibiotic levels in the defect and the controlled release profile of the biphasic bone substitute. [42] McNally et al. further reported that only 6 of 100 patients suffered from self-limiting white wound drainage, which had the appearance of liquefied calcium sulfate residue, in a prospective case series. [43] In a review of ceramic biocomposites, Ferguson et al. linked this potential reduction of wound ooze to a smaller volume of calcium sulfate and a larger percentage of less soluble HA. [37] In contrast, Hofmann et al. reported a very low complication rate in a detailed list of adverse events, with only one out of 62 patients each suffering from seroma, prolonged wound healing and implant site inflammation; Horstmann et al. observed soft tissue inflammation in 7 out of 35 patients treated with BCBS. [19, 22] Although this condition generally does not seem to require further surgical intervention, surgeons need to be aware of the possibility of soft tissue inflammation and secretion after the application of BCBS and carefully monitor the resulting surgical wounds.

## Limitations

A possible treatment bias needs to be reported, as treatment and indications, especially when choosing percutaneous surgery over open surgery, were not standardized. In general, open surgery was performed for solid tumours or recurrent cysts when metal removal surgery was additionally indicated, while percutaneous surgery was performed for cystic lesions.

As the bone cavity after BCBS treatment undergoes a constant transformation until one year after surgery, it was difficult to differentiate early recurrences and tumour progression from residual material in the postoperative cavity on X-ray and MRI. It cannot be ruled out that revision surgeries might have been undertaken, although BCBS ingrowth was not fully completed. Consequently, surgical treatment decisions were postponed to at least one year after surgery in cases of unclear or borderline cavity appearances.

Due to the heterogeneity of the underlying lesions, no direct comparisons of recurrence-free survival can be made. However, the restriction to benign bone lesions should provide a safe basis for the use of injectable synthetic allografts, especially in cases of recurrence and confirmed histology.

## Conclusion

BCBS opens further possibilities for cavity filling in percutaneous and open treatments for bone cysts as it showed good bone remodelling features in the postoperative course. However, initial wound leakage and recurrence seemed to be associated with percutaneous injection. This information helps treating surgeons identify indications for minimally invasive therapy and raises awareness for surgical site inspections. Further studies are needed to compare the recurrence and adverse event rates of open and minimally invasive bone lesion surgeries.

## Data Availability

The datasets used and/or analysed during the current study are available from the corresponding author on reasonable request.

## Abbreviations

HA: Hydroxyapatite
BCBS: Biphasic ceramic bone substitute
UBC: Unicameral bone cyst
ABC: Aneurysmal bone cyst
sABC: Secondary aneurysmal bone cyst
NOF: Non-ossifying fibroma
MRI: Magnetic resonance imaging
